# Insect pollination reduces yield loss following heat stress in faba bean (*Vicia faba* L.)

**DOI:** 10.1016/j.agee.2015.12.007

**Published:** 2016-03-15

**Authors:** Jacob Bishop, Hannah Elizabeth Jones, Martin Lukac, Simon Geoffrey Potts

**Affiliations:** a Centre for Agri-Environmental Research, School of Agriculture, Policy and Development, University of Reading, Reading, Berkshire RG6 6AR, UK; b Czech University of Life Sciences in Prague, Kamýcká 129, 165 21 Praha 6 - Suchdol, Czech Republic

**Keywords:** Faba bean, Heat stress, Pollination, Climate change, Yield stability, Yield variability

## Abstract

Global food security, particularly crop fertilization and yield production, is threatened by heat waves that are projected to increase in frequency and magnitude with climate change. Effects of heat stress on the fertilization of insect-pollinated plants are not well understood, but experiments conducted primarily in self-pollinated crops, such as wheat, show that transfer of fertile pollen may recover yield following stress. We hypothesized that in the partially pollinator-dependent crop, faba bean (*Vicia faba* L.), insect pollination would elicit similar yield recovery following heat stress. We exposed potted faba bean plants to heat stress for 5 days during floral development and anthesis. Temperature treatments were representative of heat waves projected in the UK for the period 2021–2050 and onwards. Following temperature treatments, plants were distributed in flight cages and either pollinated by domesticated *Bombus terrestris* colonies or received no insect pollination. Yield loss due to heat stress at 30 °C was greater in plants excluded from pollinators (15%) compared to those with bumblebee pollination (2.5%). Thus, the pollinator dependency of faba bean yield was 16% at control temperatures (18–26 °C) and extreme stress (34 °C), but was 53% following intermediate heat stress at 30 °C. These findings provide the first evidence that the pollinator dependency of crops can be modified by heat stress, and suggest that insect pollination may become more important in crop production as the probability of heat waves increases.

## Highlights

• Heat stress results in lower yields in faba bean.
• Insect pollination partially recovered faba bean yield loss following heat stress.
• Increased transfer of pollen to damaged flowers is the likely mechanism.
• Insect pollination may increase production stability under increasing heat stress.

## 1 Introduction

The Intergovernmental Panel on Climate Change projects that crop production and food security will be increasingly threatened this century due in part to increased climate variability, including the increased frequency and magnitude of heat waves (Kirtman et al., 2013, Porter et al., 2014, Seneviratne et al., 2012). Especially large yield losses can occur when high temperatures cause damage during crop floral development and anthesis (Hedhly, 2011, Luo, 2011), as many crop products (*e.g*., fruits, grains) are the direct result of successful fertilization. Insect pollinated crops constitute approximately a third of global food production (Klein et al., 2007), but there is no comprehensive evidence of how their fertilization may be affected by heat stress. Studies in these crops have typically measured the effect of heat stress in absence of insect pollinators (Peet et al., 1998, Young et al., 2004), potentially missing important changes in the interactions between plants and their pollinators following stress. Studies have shown that the yield of plants can be partially recovered following stress by hand provision of fertile pollen, in tomato (*Solanum lycopersicum*) (Peet et al., 1998), oilseed rape (*Brassica napus*) (Young et al., 2004), common bean (*Phaselous vulgaris*) (Gross and Kigel, 1994, Monterroso and Wien, 1990) and wheat in (*Triticum aestivum*) (Briggs et al., 1999, Saini and Aspinall, 1982). Insect pollinators may promote similar yield resilience to heat stress in entomophilous crops, through their role as pollen vectors between flowers. Such a resilience mechanism is possibly an unexpected and unquantified benefit of insect pollination, which has already been estimated to be worth $232–$577 billion each year globally (Lautenbach et al., 2012) due to increases in total crop production of 3–8% (Aizen et al., 2009). This is pertinent at a time when the threats of climate change to insect pollinator communities are becoming apparent (Carvalheiro et al., 2013).

This study investigates interactions between heat stress and insect pollination on the yield of faba bean (*Vicia faba* L.). In faba bean, vulnerability to heat stress varies between stages of floral development (Bennell et al., 2007). Therefore, heat stress at a given time point could damage some flowers while others remain undamaged through differences in the timing of their development, providing a source of fertile pollen. In a typical faba bean crop, a proportion of pollination is by spontaneous auto-fertilization, while the remainder requires an insect visit (*e.g.,* Chen, 2009). Following heat stress however, all flowers with damaged pollen would effectively be male-sterile and unable to self-pollinate (Drayner, 1959). Yield in these flowers would therefore become more dependent upon the transfer of fertile pollen by insect pollinators (yield recovery by outcrossing). The pollinator dependency of faba bean can be influenced by many factors including cultivar and location (*e.g.,* Suso et al., 2001), but under typical non-stress conditions approximately 25% of faba bean yield is dependent upon insect pollination (Ghamdi et al., 2003; Somerville, 1999). Across the majority of Europe, the most common insect pollinators of faba bean are wild bumblebees (Carré et al., 2009, Free, 1993), populations of which are projected to undergo large distribution shifts due to climate change (Kerr et al., 2015, Rasmont et al., 2015). Faba bean is already a globally important grain legume (FAO, 2015) and demand for it is likely to increase with increasing recognition of the beneficial role of faba bean in sustainable cropping (Köpke and Nemecek, 2010), the rising requirements for plant protein for both human and animal nutrition (Tilman et al., 2011), and recent policy changes that encourage multiple cropping in Europe (European Parliament News, 2013).

Using a novel experimental approach replicated over three years, we exposed potted winter faba bean plants (cultivar Wizard) to five-day temperature treatments before moving them to flight cages to be either pollinated by domesticated bumblebee colonies, or to receive no insect pollination, in order to evaluate the following hypotheses: (1) pollination by *Bombus terrestris* reduces yield mass losses following heat stress in faba bean; (2) pollination by *Bombus terrestris* reduces losses in faba bean quality (*e.g.,* mass per bean, protein content) following heat stress; (3) observed changes in yield can be attributed to changes in fertilization (*e.g*., bean number) following insect pollination.

## 2 Methods

### 2.1 Experimental design and growing conditions

Experiments were conducted over three growing seasons from 2012 to 2014 at the Plant Environment Laboratory (now succeeded by the Crop and Environment Laboratory), University of Reading, UK. All experimentation (Table 1) was designed to test whether insect pollination modifies the response of potted winter faba bean (*Vicia faba* L.) to heat stress during floral development and anthesis. Plants were exposed to temperature treatments for five days during early flowering (Table S1, Supplementary material) and subsequently moved to flight cages where they were either exposed to a colony of domesticated bumblebees or received no insect pollination.

We used the synthetic cultivar, Wizard (Wherry & Sons Ltd.), a UK recommended list commercial cultivar since 2003 (PGRO, 2015). Plants were randomly assigned to temperature treatments and flight cages in all experiments. All experimental plants were grown in plastic pots (180 mm diameter; 4 l volume) containing vermiculite, sand, gravel and compost at a ratio of 4:2:4:1, mixed with 2 kg m^−3^ Osmocote slow-release granules (LBS Horticulture Ltd.). Three seeds were sown per pot, allowing thinning to one plant per pot when 3 leaf pairs had unfolded on the majority of plants. Plants were maintained in a fully enclosed polytunnel until on average 4 leaf pairs had unfolded on each plant, when they were moved and randomly distributed either in the open (2012) or within flight cages (2013 and 2014) until temperature treatments. Plants were watered to maintain field capacity throughout experiments including during temperature treatments, at least daily by hand watering in 2012, and drip-irrigation in 2013 and 2014. Three consecutive replicate experiments were conducted in 2013 over a period of 18 days (Table 1), and plants were manually assigned to replicates to standardise developmental stage.

### 2.2 Temperature treatment

Five temperature treatments (18/10, 22/14, 26/18, 30/22, 34/26 °C day/night temperature) were chosen to measure responses over a wide range of potential temperature anomalies, and because there was no prior information about heat stress vulnerability of faba bean. Temperature treatments 26, 30 and 34 °C were intended to represent heat wave scenarios that are projected to be common during the period 2021–2050 in the UK and Western Europe (Fischer and Schär, 2010), with 30 and 34 °C in particular representing levels of stress that may occur through combinations of high temperatures and reduced soil moisture (Alghabari et al., 2014, Lobell et al., 2011). All treatments comprised transferring plants from flight cages at midday to five 1.37 × 1.47 m^2^ Saxcil growth cabinets for a duration of five days during early flowering (Table S1, Supplementary material). The photoperiod lasted 16 h and the transition between night and day temperatures took approximately 15 min. Conditions were monitored throughout temperature treatments; light levels were maintained at 650 μmol photon m^−2^ s^−1^; relative humidity was 87 ± 13% in 2012, 80 ± 20% in 2013 and 85 ± 15% in 2014; and CO_2_ was 385 mg L^−1^. Temperature was measured by a thermocouple at pot height. Growth cabinet temperatures were randomly reassigned between years and during 24 h between replicate experiments in 2013.

### 2.3 Pollination treatment

Following temperature treatments, plants were moved to flight cages (Table 1) which were used to either retain single domesticated colonies of *Bombus terrestris audax* L. (a common wild visitor of faba bean in the field; Garratt et al., 2014) that were applied following temperature treatments, or to completely prevent visits from insect pollinators. While this method does not represent a typical pollinator community visiting faba bean in the field, it enables a controlled comparison between pollination treatments without confounding effects of bagging that could otherwise modify plant growth and yield accumulation in excluded plants (Free, 1993). All cages were custom-made (Lancashire Sports Repair) from 1.33 mm^2^ aperture polyethylene mesh (WM16, Wondermesh). In each year, all treatment cages were within an area of 12.5 × 5 m. Following common practise in reciprocal outcrossing experiments (*e.g.,* Saini and Aspinall, 1982), experimental plants were housed with non-stressed pollen donor individuals to ensure provision of fertile pollen. The ratio of pollen donor to experimental plants was 3:1 in 2012, but was later reduced to 1:1 following an additional experiment which demonstrated this was a sufficient ratio to achieve good pollination (data not shown). Experimental plants that had been exposed to different temperatures were housed together in the same flight cage; thus maintaining the validity of temperature treatment comparisons. In 2013, flight cages were repeatedly allocated to the same pollination treatment across the three replicate experiments, but were analysed as independent replicates because a new *B. terrestris* colony was used each time. To standardise timing of pollinator exposure across all experiments, in 2013 the pollination treatment plants assigned to the third replicate experiment were held in the exclusion cage, while replicate two plants were exposed to stress, and replicate one plants received insect pollination.

### 2.4 Data collection

Yield parameters were assessed when plants had reached senescence. Pods on all experimental plants were individually harvested with node and raceme position recorded, to allow changes in within-stem yield allocation to be investigated. Pods were oven dried at 80 °C until dry mass was constant before recording bean mass. Bean number was measured using WINDIAS image analysis software (version 3, Delta T Devices), recorded to whole plant level in 2012 and pod-level in 2013 and 2014.

Yield mass per plant was calculated for all years, by summing the mass of beans produced by pods on each plant. The yield mass benefit due to insect pollination was calculated for each temperature treatment level, by dividing the average per-plant yield of an insect pollination cage by that of the exclusion cage, in each year, or replicate experiment in 2013. The 10 cages used in 2014 were randomly allocated to treatments and therefore not paired, so for 2014 the combined means of all cages containing bees and those excluding pollinators were compared, the statistical analysis was weighted accordingly. Mass per bean, and the number of beans per pod, were calculated by averaging across pods within each plant. Changes in yield allocation on the primary stem were tested using the first node to set pods on each plant. The yield ratio was measured by dividing yield mass by the mass of stems (with leaf and raceme branches removed) and pod casings for each plant in 2014. Seed nitrogen content per plant, as a proxy for protein content, was measured on a subset of plants in 2013 (150 plants) and 2014 (100 plants) using a LECO FP-328 analyser.

### 2.5 Statistical analysis

Plant level yield parameters (yield mass, bean number, pod number (data from all years); bean number per pod, mass per bean, first node with pod, nitrogen content (2013 and 2014), yield ratio, non-yield biomass (2014 only)) were analysed with linear mixed effects models (Table S3, Supplementary material) via the lme4 package (Bates et al., 2014) in R statistical software (version 3.2.0, R Core Team 2015). Repeated measures of multiple plants within each cage, and differences in the number of replicate plants between years, were addressed by the random effect (1|cage). Temperature treatments were analysed as a categorical factor, to allow for simpler analysis and interpretation of complex non-linear relationships between temperature and pollination treatments. Plants within each cabinet were treated as independent replicates of a temperature treatment; the temperature treatment was the dominant factor affecting plants within each cabinet, and cabinets were randomly allocated to different temperature treatments between replicated experiments in 2013, and across years. Yield parameters that were calculated on a larger than plant level (yield benefit of pollination; yield variability), were analysed with ANOVA using the means of plants from each combination of flight cage and cabinet (Table S3, Supplementary material). Analysis of yield benefit due to pollination included a weighting term (5 times higher weighting for 2014), as the single figure for 2014 was derived from 5 comparisons of cages containing and excluding insect pollinators. Year was considered a fixed effect in all models to assess the between-year variability.

To establish the effect of treatments on yield parameters (Table S3, Supplementary Material), maximal models, containing parameters: temperature, pollination, interaction of temperature and pollination, and year, were simplified by single term deletions tested with likelihood ratio tests (Shmueli, 2010). Single terms were dropped if *p* > 0.05. After all single term deletion tests had been performed, temperature treatment levels with similar model predicted estimates were grouped for simplicity of interpretation (Crawley, 2013), provided model explanatory power was not reduced (*p* > 0.1). Model residuals were checked for normality and heteroscedasticity, yield ratio was exponential-transformed and yield variability was square-root transformed to improve model fit. Effect sizes provided in the text are model parameter estimates, raw data values are provided in the figures and Table 2.

## 3 Results

### 3.1 Yield parameters

Whole-plant yield and the yield benefit attributable to insect pollination were analysed to understand the response of faba bean plants to insect pollination following heat stress.

#### 3.1.1 Per plant yield

**Fig. 1:**
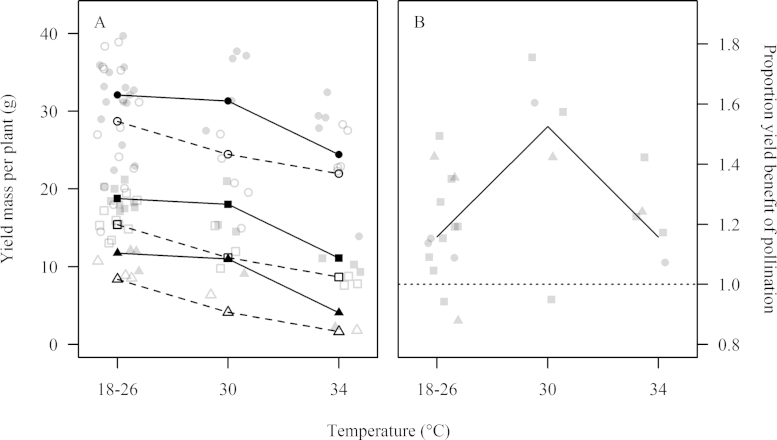
Responses of yield parameters to heat stress and pollination treatments. Point styles represent pollination treatment and year, filled points = insect pollination; open points = exclusion; triangles = 2012; squares = 2013; circles = 2014. Points are jittered horizontally to aid viewing. A: Yield mass per plant. Lines represent model estimated means for each temperature category, for insect pollinated plants (solid line) or plants excluded from pollination (dashed line); B: Proportion of yield attributable to insect pollination (yield mass of insect pollinated plants/excluded plants). Line represents model estimated mean for each temperature category, dashed line indicates level at which yield mass of insect pollinated and excluded plants are equal.

The response of whole-plant yield to heat stress (Fig. 1A) was significantly modified by pollination (*p* = 0.036). Following the 30 °C temperature treatment the yield of plants grown in cages without bees was reduced by 4.2 g per plant (at least 15%), while the yield of insect-pollinated plants was reduced by 0.8 g (at least 2.5%) compared to control temperatures. Yields of both insect-pollinated and excluded plants were reduced following the 34 °C temperature treatment, with reductions of 7.6 g and 6.7 g compared to the respective control treatments. The heat wave scenario treatment of 26 °C did not significantly differ from control temperatures 18 and 22 °C, so these temperatures were grouped as one control level (*p* = 0.539) after significance of the treatments had been established.

#### 3.1.2 Yield benefit from pollination

In addition to modifying the relationship of yield and heat stress in terms of absolute yield values, the proportional yield benefit attributable to insect pollination (Fig. 1B) increased from 15.8% under control temperatures (18, 22 and 26 °C; grouping *p* = 0.591) to 52.5% following the 30 °C heat stress treatment (*p* = 0.004). Following exposure to 34 °C, however, the benefit of pollination (15.8%) was identical to control temperatures.

### 3.2 Fertilization and yield quality parameters

The number of beans per pod and per plant were analysed to assess changes in fertilization success. To explore the mechanisms by which pollinators modified yield and their impact on yield quality, yield allocation; yield ratio; yield variability; and mass of individual beans were analysed.

#### 3.2.1 Bean and pod number

Bean number per plant (Table 2) was not affected by an interaction between temperature and pollination treatments (*p* = 0.117), however, temperature treatments of 30 and 34 °C (18 to 26 °C were grouped, *p* = 0.101) reduced bean number by 6.6 and 14.7 respectively (*p* < 0.001), and plants excluded from insect pollinators produced on average 6.9 (at least 12%) fewer beans. Bean number per pod (Table 2) was affected by an interaction between heat stress and pollination (*p* < 0.001), each level of temperature was significantly different. Pod number per plant (Table 2) was not affected by insect pollination (*p* = 0.386), but was reduced following the 30 and 34 °C treatments (*p* < 0.001).

#### 3.2.2 Yield ratio and within-plant yield allocation

**Fig. 2:**
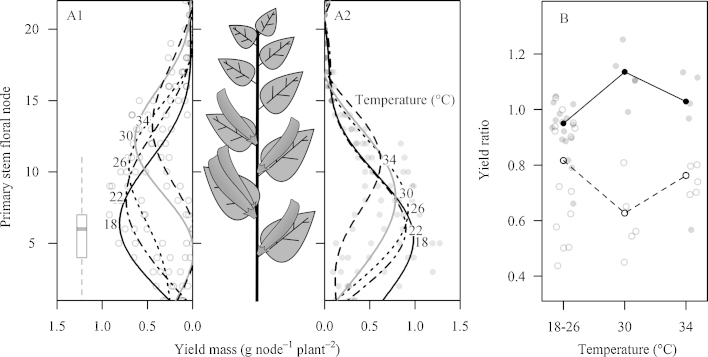
Point styles represent pollination treatment and year, open points = exclusion (panel A1); filled points = insect pollination (panel A2). A: Distribution of yield on the primary stems of experimental plants in 2013 and 2014; lines are model predictions from generalised additive models restricted to 5 basis dimensions to produce readily comparable model fits, of the average yield mass per node per plant for separate heat stress treatments. Line styles represent different temperature treatments. Boxplot shows number of floral nodes on main stems with flowers present (counts included un-opened flowers at green bud stage) prior to temperature treatments, across all treatments. B: Yield ratio of plants in 2014. Points are jittered horizontally to aid viewing. Lines represent model estimated mean for each temperature category.

The first node to set pods moved away from those flowers present prior to stress with temperature (Fig. 2A) and was 5.5 and 7.8 nodal positions higher following 30 and 34 °C temperature treatments in plants excluded from insect pollination, while smaller changes of 1.9 and 3.8 nodes were measured in pollinated plants (*p* = 0.005), each level of temperature was significantly different. Insect pollinated plants produced around 3 g less non-yield biomass (Table 2) per plant (*p* = 0.030) and non-yield biomass was also reduced by an average of 3.5 g per plant across both pollination treatments following the 30 and 34 °C temperature treatments (*p* = 0.001). There was no interaction between temperature and pollination (*p* = 0.389) and no significant difference between the two hottest treatments (*p* = 0.126). Yield ratio (Fig. 2B) of insect pollinated plants was approximately 20% higher following the 30 °C temperature treatment (interaction term; *p* = 0.001).

#### 3.2.2 Yield variability

The yield of plants within a combination of temperature treatment and flight cage was approximately 18% less variable in cages that contained bees, than in cages without bees (Table 2; *p* = 0.021). The coefficient of variation (standard deviation/mean) was unaffected by temperature treatments (*p* = 0.488) but changed between years of experimentation (*p* < 0.001). Other yield parameters changed between years; total yield mass per plant (*p* < 0.001), bean number per pod (*p* < 0.001) and per plant (*p* < 0.001) all differed between years, while the proportional benefit of pollination remained stable between years (*p* = 0.784).

#### 3.2.3 Mass per bean and nitrogen content.

Thousand grain weight (*i.e.,* individual bean mass × 1000) of insect pollinated plants increased by 45 and 55 g following the 30 and 34 °C temperature treatments from 460.15 g at control temperatures, compared to an increase of 31 g and a decrease of 52 g measured in plants excluded from pollinators (interaction term; *p* = 0.020). Percentage nitrogen content was 0.18 higher following the 26, 30 and 34 °C temperature treatments (*p* = 0.039) and differed with year (*p* = 0.032), though these differences are small and equate to around a one percent change in protein content.

## 4 Discussion

The main aim of this study was to investigate interactions between heat stress and insect pollination on the yield of faba bean. Our results suggest that sufficiently pollinated faba bean crops could have less variable yields that are more resilient to heat stress. We measured an increase in the pollinator-dependency of experimental plants with heat stress, from 16% dependency at control temperatures, to 53% dependency in plants exposed to 30 °C treatment, before dropping back to 16% dependency at 34 °C. This change in the benefit of insect pollination occurred because following heat stress at 30 °C, yield losses of at least 15% occurred in plants that were excluded from pollinators, while significantly lower yield losses occurred in plants that were pollinated by *Bombus terrestris*. At 34 °C, female floral organs may have been damaged to the point that fertilization was not possible, or other processes such as plant vegetative growth may have been affected so that bee-dependent yield recovery could not be realised. Enhanced yield resilience to stress was a previously unknown benefit of insect pollination. Experiments to compare the vulnerability of male and female floral organs have however measured similar yield recovery following stress and the manual transfer (*e.g.,* by hand) of fertile pollen in tomato (Peet et al., 1998), oilseed rape (Young et al., 2004), common bean (Gross and Kigel, 1994, Monterroso and Wien, 1990), and wheat (Briggs et al., 1999, Saini and Aspinall, 1982). This suggests that there is potential for pollination to mitigate the negative effects of heat stress on productivity of other insect-pollinated crops. It is interesting that yield increased during the three years of our experimentation, this was likely due to continuous optimisation of growth conditions of our potted plants. The benefit to yield or yield stability provided by insect pollination was conserved across the range of faba bean productivity.

It is not clear from our experiment whether insect pollinators actually improved yield resilience to heat stress by moving fertile pollen to pollen-deficient flowers (yield recovery by outcrossing). In faba bean, a floral visit can either lead to outcrossing, or can facilitate within-flower self-pollination by disrupting (tripping) a physical barrier between the stigma and anthers that otherwise prevents self-pollination in some flowers (Kambal et al., 1976). Insect pollination may have simply facilitated greater levels of self-pollination in flowers that were less damaged by the stress treatment. The number of beans per plant, arguably a more direct measure of fertilization, was not augmented by insect pollination to the same extent as yield mass. However, yield allocation was retained on lower, more productive floral nodes following heat stress in insect pollinated plants (and was retained closer to flowers present prior to stress), while yield at these nodes was lost in excluded plants. This may have promoted yield resilience through changes in resource use efficiency, which increased dramatically following the 30 °C treatment in insect pollinated plants, contrasting with a reduction in excluded plants. Confirming the mechanism by which resilience occurred is important to effectively target interventions. We studied a single cultivar to control differences in outcrossing, but resilience could be higher in certain faba bean cultivars that increase outcrossing rate through *e.g.,* high floral attractiveness to pollinators (Suso et al., 2005). If resilience is due to the increased outcrossing following heat stress, this could be established using a genetic approach (*e.g.,* Ritland and Jain, 1981).

To understand the importance of beneficial interactions that we observed, it is useful to quantify the likelihood of extreme temperatures occurring during crop floral development and anthesis. However, while there is consensus among projections that heat waves are likely to become hotter and more frequent in the future (Donat and Alexander, 2012, Hansen et al., 2012, Kirtman et al., 2013, Seneviratne et al., 2012), projecting the absolute temperatures and timing of extreme events remains problematic and susceptible to bias (Seneviratne et al., 2012). Available projections for the UK suggest that heat waves (≥6 consecutive days with peak temperature ∼26 °C) will increase from approximately a 1 in 5 year to a 1 in <2 year occurrence in summer months of the period 2021–2050 (Fischer and Schär, 2010), occurrences of rarer, hotter, heat waves are more difficult to predict and were not provided. Furthermore, directly relating our experimental temperature treatments to climate change scenarios relies on at least two other assumptions, (i) that atmospheric carbon dioxide concentrations [CO_2_] will not increase, or affect yield resilience, (ii) that soil moisture will not limit plant evapotranspiration. Future [CO2] emissions greatly depend upon human actions, and impacts of increased [CO2] on crop production are variable (Ainsworth and Long, 2005). Drought is projected to increase in the future (Kirtman et al., 2013), so the temperature treatments of 30 and 34 °C may represent stress levels that plants will experience at lower temperatures, if combined with low soil moisture (*e.g.,* ‘compound events’; Seneviratne et al., 2012). Experimental plants were well watered and evaporative cooling undoubtedly increased the temperature at which yield reductions occurred (Alghabari et al., 2014, Lobell et al., 2011). Further work is required to quantify the relative likelihoods of stress levels represented by the 30 and 34 °C treatments, to understand how frequently faba bean pollinator dependency will increase above typical levels.

The average yield benefit of insect pollination of approximately 16% that we measured at control temperatures falls within the range of other studies comparing faba bean plants in cages with and without insect pollinators *e.g.,* 15% (Garratt et al., 2014); 26% (Ghamdi and Ghamdi, 2003) and 25% (Somerville, 1999). Higher reported benefits may be due to varietal differences, plant stress, or detrimental effects of bagging in experiments that compared yields of bagged plants with openly pollinated controls (Benachour et al., 2007, Free, 1993, Nayak et al., 2015). We found additional benefits of pollination across all tested temperatures, in agreement with existing literature, pollination increased the number of beans per plant (Ghamdi and Ghamdi, 2003) and per pod (Garratt et al., 2014) indicating that improved fertilization enabled allocation of yield on lower nodes (Somerville, 1999, Suso et al., 1996). This can reduce lodging risk and improve uniformity of ripening (Stoddard, 1993), but did not affect seed nitrogen content (Bartomeus et al., 2014). Between-plant variability was high in all experiments but insect pollination reduced this variability in yield across all temperature treatments. This is of high importance as yield variability is a key concern for faba bean growers (*e.g.,* Rubiales, 2010).

Our findings provide robust evidence that insect pollinators can elicit partial yield compensation following stress in faba bean, and therefore that pollinator dependency of faba bean and other self-compatible crops may increase with greater likelihood of heat stress during flowering. Our experimental methodology assumed that insect pollinators will be present, and able to provide this yield resilience benefit in the future. However, the current literature suggests that pollinator communities will be strongly affected by climate change (Kerr et al., 2015, Polce et al., 2014, Rasmont et al., 2015). More research is required to help understand (and mitigate) the threats of both gradual climate change on pollinator populations, and the effects of extreme weather on floral visitation by insect pollinators. With an eroded pollinator population in the future, methods to improve the interactions of crop plants and their pollinators (*e.g.,* Garibaldi et al., 2014) will be further necessitated. In faba bean, evidence suggests that pollination services are higher and more stable when fields are closer to semi-natural habitats (Andersson et al., 2014, Garibaldi et al., 2011, Garratt et al., 2014, Nayak et al., 2015; but see Bartomeus et al., 2014). In landscapes where the natural pollinator community has been degraded, provision of managed pollinators to supplement wild pollinators may be the only feasible option to improve crop pollination. Supplementation with honeybees (*Apis mellifera*) can enhance yield (Stoddard, 1986) and has been shown to be economically viable in Australia (Cunningham and Le Feuvre, 2013). Further work is required to quantify the density and diversity of pollinators necessary to achieve optimal pollination in faba beans and also to determine whether the beneficial interactions that we measured occur in field conditions with a wild pollinator community. Beneficial interactions may be achieved with fairly low pollinator numbers; a study that controlled pollinator visits to individual flowers found no effect of visit number on pod set (Garratt et al., 2014).

This study was novel in exploring interactions between abiotic stress and insect pollination and their effects on crop yield production. In our experimental system, caged *Bombus terrestris* colonies contributed to a significant proportion of faba bean yield under all temperature treatments, and mitigated observed reductions in yield mass and some yield quality parameters (yield ratio, individual bean mass) following the 30 °C heat stress treatment. Yield production became dramatically more dependent on insect pollination following the 30 °C treatment, suggesting that insect pollination may become increasingly important with increasing incidence of heat stress. The potential impacts of this could be great in less developed countries where climate change is expected to have disproportionately large effects for food security (Porter et al., 2014) and where the cultivation of pollinator-dependent crops is higher (Aizen et al., 2009). Given that 75% of global crops benefit from insect pollination (Klein et al., 2007) it is important to understand how widespread this phenomenon is for production stability. Our findings highlight the importance of understanding the threats to and conserving key pollinating species that may improve the resilience of crop production to projected climate change, in order to promote both current and future food security.

## Figures and Tables

**Table 1:** Summary of experimental designs, treatment combination refers to an individual combination of flight cage and controlled environment cabinet.

Year	Sow date	Plant number		Replicate experiments	Flight cage specifications			
Total	Per treatment combination	Location (lat, long)	Number	Dimensions (m)	Donor: experimental plant ratio
2012	8 Dec 11	100	10	1	Sonning Farm (51 48′ N, 00 89′ W)	2	2.4 × 2.4 × 2.1	3:1
2013	11 Jan 13	190 (570)	19 (57)	3	Plant Environment Lab (51 27′ N, 00 56′ W)	2 (6)	12.5 × 2.5 × 2.5	1:1
2014	13 Jan 14	200	4	1	Plant Environment Lab (51 27′ N, 00 56′ W)	10	2.5 × 2.5 × 2.5	1:1

**Table 2:** Absolute yield parameter values aggregated across experimental years and cages. Test statistics and *p* values (bold values are significant to *p *< 0.05) provided are from likelihood ratio tests; *χ^2^* tests for mixed models or *F* tests for linear models, between candidate models following single-term deletions.

Treatments		Parameters (mean ± SEM)						
Temperature (day/night; °C)	Pollination	Bean number	Pod number	Beans per pod	Mass per bean (g)	Yield mass variability	% Nitrogen	Non-yield biomass (g)
18/10	Pollinated	51.9 ± 5.2	17.2 ± 1.8	2.9 ± 0.1	0.510 ± 0.014	0.378 ± 0.080	4.295 ± 0.098	35.848 ± 1.953
Exclusion	42.8 ± 5.8	15.5 ± 6.7	2.5 ± 0.1	0.534 ± 0.018	0.425 ± 0.062	4.542 ± 0.066	37.566 ± 2.164
22/14	Pollinated	46.0 ± 4.5	15.4 ± 2.5	2.6 ± 0.2	0.521 ± 0.014	0.414 ± 0.047	4.398 ± 0.110	34.162 ± 0.927
Exclusion	40.3 ± 4.7	15.6 ± 7.6	2.5 ± 0.1	0.528 ± 0.019	0.430 ± 0.076	4.469 ± 0.143	34.018 ± 1.912
26/18	Pollinated	48.3 ± 4.6	17.0 ± 3.8	2.7 ± 0.0	0.494 ± 0.013	0.322 ± 0.054	4.560 ± 0.087	32.993 ± 1.299
Exclusion	42.0 ± 5.1	16.8 ± 8.8	2.4 ± 0.1	0.565 ± 0.021	0.403 ± 0.075	4.624 ± 0.115	36.232 ± 1.419
30/22	Pollinated	43.7 ± 4.8	17.5 ± 4.1	2.5 ± 0.1	0.591 ± 0.017	0.307 ± 0.049	4.512 ± 0.130	30.723 ± 1.988
Exclusion	31.8 ± 3.0	15.0 ± 9.7	2.0 ± 0.0	0.556 ± 0.020	0.496 ± 0.084	4.559 ± 0.090	35.238 ± 1.064
34/26	Pollinated	32.8 ± 5.2	14.5 ± 5.2	2.1 ± 0.1	0.608 ± 0.023	0.432 ± 0.094	4.539 ± 0.089	27.445 ± 0.654
Exclusion	31.5 ± 5.4	15.0 ± 10.6	2.1 ± 0.0	0.552 ± 0.022	0.570 ± 0.136	4.517 ± 0.078	33.135 ± 0.760

Treatment effects								
Interaction Pollination: temperature		*χ^2^* = 5.671; *p* = 0.117	*χ^2^* = 3.441; *p* = 0.487	*χ^2^* = 26.91; *p* < **0.001**	*χ^2^* = 7.873; *p* = **0.005**	*F* = 0.703; *p* = 0.593	*χ^2^* *=* *6.7102; p* = 0.152	*χ^2^* = 4.126; *p* = 0.389

Pollination		*χ^2^* = 5.178; *p* = **0.023**	*χ^2^* = 0.753; *p* = 0.386	*–*	*–*	*F* = 5.508; *p* = **0.021**	*χ^2^* *=* 0.6945*; p* = 0.405	*χ^2^* = 4.725; *p* = **0.030**

Temperature		*χ^2^* = 118.84; *p* < **0.001**	*χ^2^* = 33.175; *p* < **0.001**	*–*	*–*	*F* = 0.865; *p* = 0.488	*χ^2^* *=* 10.100*; p* = **0.039**	*χ^2^* = 16.181; *p* = **0.003**

Year		*χ^2^* = 25.002; *p *< **0.001**	*χ^2^* = 33.680; *p* < **0.001**	*χ^2^* = 28.625; *p* < **0.001**	*χ^2^* = 13.845; *p* < **0.001**	*F* = 21.489*;* *p* < **0.001**	*χ^2^* *=* 4.612*; p* = **0.032**	*–*

Simplified temperature categories		18-26, 30, 34	18-26, 30, 34	**–**	18-26, 30, 34	**–**	18-22; 26-34	18-26, 30-34
