# Supplementary material for: Insect pollination reduces yield loss following heat stress in faba bean (Vicia faba L.)
Source: Agric Ecosyst Environ. 2016 Mar 15;220:89–96. doi: 10.1016/j.agee.2015.12.007 (PMC4767028; doi:10.1016/j.agee.2015.12.007)
Supplement: Supplementary file 1 [file mmc1.docx]

**Insect pollination reduces yield loss following heat stress in faba bean (*Vicia faba* L.).**

J. Bishop*, H.E. Jones, M. Lukac, S.G. Potts.

Centre for Agri-Environmental Research, School of Agriculture, Policy and Development, University of Reading, Reading, RG6 6AR, UK

**Supplementary Online Material**

**Table S1:** Number of floral nodes with flowers identified as either present or open on the main stems of experimental plants, immediately prior to temperature treatments.

| **Year** | **Replicate** | **No. floral nodes on main stem with flowers present (e.g. including flowers at green-bud stage); mean ± SD** | **No. floral nodes on main stem with flowers open (open flowers only); mean ± SD** |
| --- | --- | --- | --- |
| 2012 | 1 | 3.2 ± 1.8 | 0.5 ± 0.9 |
| 2013 | 2 | 5.6 ± 1.3 | 1.9 ± 1.1 |
|  | 3 | 6.2 ± 1.4 | 1.5 ± 1.5 |
|  | 4 | 6.0 ± 1.5 | 2.4 ± 1.0 |
| 2014 | 5 | 4.0 ± 2.1 | 0.4 ± 0.8 |

**Table S2:** Explanation of model parameters.

| **Parameter** | **Explanation** |
| --- | --- |
| heatcat | temperature treatment, categorical |
| poll | pollination treatment |
| heatcat:poll | interaction between temperature and pollination treatments |
| year | year of experimentation |
| 1\|cage | random effect, fitting separate intercepts for each cage |

**Table S3:** Model simplification tables for establishment of treatment effects and effect sizes. Model parameters are deleted from the maximal model in steps, if a likelihood ratio test comparing the new model with the previous model finds no significant difference in explanatory power (p>0.05), the parameter is left out of the model. Simplification of heat levels is conducted after all other treatment effects have been established, *p*>0.1 indicates no significant difference in explanatory power between a previous model containing all heat levels, and a new model containing the presented heat categories. Mixed models are distinguished by a random effect (1|cage) in the maximal model, candidate mixed models are compared with a **χ**^2^ test and linear models compared with an *F* test. Highlighted values are those included in the text, green highlights indicate a significant difference (resulting in no subsequent deletion of that parameter), yellow highlights indicate a non-significant difference in model explanatory power (resulting in the deletion of that parameter from subsequent models).

1. **Yield mass per plant**

| **Maximal model:** | outpod~heatcat+poll+heatcat:poll+year+(1\|cage) | | | | | | | |
| --- | --- | --- | --- | --- | --- | --- | --- | --- |
| **Deletion step** | **Parameter** | **χ^2^** | | **df** | | ***p*-value** | |  |
| **1** | heatcat:poll | 10.296 | | 4 | | 0.036 | |  |
|  | year | 36.413 | | 2 | | <0.001 | |  |
| **2** | heatcat -> 18-26,30,34 | 3.1161 | | 4 | | 0.539 | |  |
| **Fixed effect in minimal adequate model** | | | **Estimate** | | **SE** | | ***t*-value** | |
| (Intercept) | | | 11.7517 | | 2.4722 | | 4.754 | |
| heatcat30 | | | -0.7557 | | 0.9058 | | -0.834 | |
| heatcat34 | | | -7.6757 | | 0.9098 | | -8.437 | |
| pollnone | | | -3.3725 | | 1.6427 | | -2.053 | |
| year2012-13 | | | 7.0208 | | 2.6671 | | 2.632 | |
| year2013-14 | | | 20.3304 | | 2.5762 | | 7.892 | |
| heatcat30:pollnone | | | -3.4893 | | 1.2924 | | -2.7 | |
| heatcat34:pollnone | | | 0.9395 | | 1.2892 | | 0.729 | |

**B) Proportion yield benefit from pollination**

| **Maximal model:** | prop~heatcat+year, weights=c(1,1,1,1,5) | | | | | | |  |  |
| --- | --- | --- | --- | --- | --- | --- | --- | --- | --- |
| **Deletion step** | **Parameter** | | ***F*** | | ***p*-value** | |  |  |  |
| **1** | heatcat | | 4.9982 | | 0.007 | |  |  |  |
|  | year | | 0.2462 | | 0.784 | |  |  |  |
| **2** | heatcat | | 5.4057 | | 0.004 | |  |  |  |
| **3** | heatcat -> 18-26,30,34 | | 0.5388 | | 0.592 | |  |  |  |
| **Fixed effect in minimal adequate model** | | **Estimate** | | **SE** | | ***t*-value** | | | ***p*-value** |
| (Intercept) | | 1.158459 | | 0.040866 | | 28.348 | | | <0.000 |
| heatcat30 | | 0.366416 | | 0.081732 | | 4.483 | | | 0.000185 |
| heatcat34 | | -0.000213 | | 0.081732 | | -0.003 | | | 0.99794 |

**C) Bean number per plant**

| **Maximal model:** | beannum~heatcat+poll+heatcat:poll+year+(1\|cage) | | | | | | | | |
| --- | --- | --- | --- | --- | --- | --- | --- | --- | --- |
| **Deletion step** | **Parameter** | **χ^2^** | | **df** | | ***p*-value** | |  |  |
| **1** | heatcat:poll | 7.3851 | | 4 | | 0.117 | |  |  |
|  | year | 24.978 | | 2 | | <0.001 | |  |  |
| **2** | heatcat | 118.84 | | 4 | | <0.001 | |  |  |
|  | poll | 5.178 | | 1 | | 0.023 | |  |  |
|  | year | 24.99 | | 2 | | <0.001 | |  |  |
| **3** | heatcat -> 18-26,30,34 | 4.5807 | | 2 | | 0.101 | |  |  |
| **Fixed effect in minimal adequate model** | | | **Estimate** | | **SE** | | ***t*-value** | |  |
| (Intercept) | | | 28.643 | | 5.023 | | 5.702 | |  |
| heatcat30 | | | -6.675 | | 1.362 | | -4.901 | |  |
| heatcat34 | | | -14.707 | | 1.359 | | -10.822 | |  |
| pollnone | | | -6.907 | | 3.249 | | -2.126 | |  |
| year2012-13 | | | 11.915 | | 5.433 | | 2.193 | |  |
| year2013-14 | | | 29.256 | | 5.254 | | 5.568 | |  |

**D) Bean number per pod, per plant**

| **Maximal model:** | beanperpod~heatcat+poll+heatcat:poll+year+(1\|cage) | | | | | | | | |
| --- | --- | --- | --- | --- | --- | --- | --- | --- | --- |
| **Deletion step** | **Parameter** | **χ^2^** | | **df** | | ***p*-value** | |  |  |
| **1** | heatcat:poll | 26.91 | | 4 | | <0.001 | |  |  |
|  | year | 28.625 | | 5 | | <0.001 | |  |  |
| **2** | heatcat -> 18-26,30,34 | 9.5917 | | 4 | | 0.048 | |  |  |
| **Fixed effect in minimal adequate model** | | | **Estimate** | | **SE** | | ***t*-value** | |  |
| (Intercept) | | | 3.04106 | | 0.11605 | | 26.206 | |  |
| heatcat22 | | | -0.17781 | | 0.093 | | -1.912 | |  |
| heatcat26 | | | -0.20017 | | 0.09338 | | -2.144 | |  |
| heatcat30 | | | -0.41861 | | 0.09331 | | -4.486 | |  |
| heatcat34 | | | -0.97548 | | 0.09383 | | -10.396 | |  |
| pollnone | | | -0.37826 | | 0.14009 | | -2.7 | |  |
| year2013-14 | | | -0.11834 | | 0.11363 | | -1.042 | |  |
| heatcat22:pollnone | | | 0.06671 | | 0.13168 | | 0.507 | |  |
| heatcat26:pollnone | | | 0.01733 | | 0.13217 | | 0.131 | |  |
| heatcat30:pollnone | | | -0.03015 | | 0.13231 | | -0.228 | |  |
| heatcat34:pollnone | | | 0.55747 | | 0.13249 | | 4.208 | |  |

**E) Pod number per plant**

| **Maximal model:** | podnum~heatcat+poll+heatcat:poll+year+(1\|cage) | | | | | | | | |
| --- | --- | --- | --- | --- | --- | --- | --- | --- | --- |
| **Deletion step** | **Parameter** | **χ^2^** | | **df** | | ***p*-value** | |  |  |
| **1** | heatcat:poll | 3.4412 | | 4 | | 0.487 | |  |  |
|  | year | 34.336 | | 2 | | <0.001 | |  |  |
| **2** | heatcat | 3.169 | | 4 | | <0.001 | |  |  |
|  | poll | 0.7532 | | 1 | | 0.386 | |  |  |
|  | year | 34.332 | | 2 | | <0.001 | |  |  |
| **3** | heatcat | 33.175 | | 4 | | <0.001 | |  |  |
|  | year | 33.68 | | 2 | | <0.001 | |  |  |
| **4** | heatcat -> 18-26,30,34 | 1.7168 | | 2 | | 0.424 | |  |  |
| **Fixed effect in minimal adequate model** | | | **Estimate** | | **SE** | | ***t*-value** | |  |
| (Intercept) | | | 7.7443 | | 1.4325 | | 5.406 | |  |
| heatcat30 | | | -0.541 | | 0.4886 | | -1.107 | |  |
| heatcat34 | | | -2.7545 | | 0.4875 | | -5.65 | |  |
| year2012-13 | | | 5.3944 | | 1.6298 | | 3.31 | |  |
| year2013-14 | | | 12.7898 | | 1.589 | | 8.049 | |  |

**F) Lowest node to set pod, per plant**

| **Maximal model:** | minimum node~heatcat+poll+heatcat:poll+year+(1\|cage) | | | | | | | | |
| --- | --- | --- | --- | --- | --- | --- | --- | --- | --- |
| **Deletion step** | **Parameter** | **χ^2^** | | **df** | | ***p*-value** | |  |  |
| **1** | heatcat:poll | 14.9 | | 4 | | <0.005 | |  |  |
|  | year | 0.0012 | | 1 | | 0.973 | |  |  |
| **2** | heatcat:poll | 14.899 | | 4 | | <0.005 | |  |  |
| **3** | heatcat -> 18-26,30,34 | 51.394 | | 6 | | <0.001 | |  |  |
|  | heatcat -> 18-22,26,30,34 | 5.0937 | | 2 | | 0.078 | |  |  |
| **Fixed effect in minimal adequate model** | | | **Estimate** | | **SE** | | ***t*-value** | |  |
| (Intercept) | | | 3.6451 | | 0.6077 | | 5.998 | |  |
| heatcat22 | | | 0.9418 | | 0.5455 | | 1.726 | |  |
| heatcat26 | | | 1.6041 | | 0.5509 | | 2.912 | |  |
| heatcat30 | | | 1.8937 | | 0.5489 | | 3.450 | |  |
| heatcat34 | | | 3.7774 | | 0.5607 | | 6.737 | |  |
| pollnone | | | 2.1975 | | 0.8613 | | 2.551 | |  |
| heatcat22:pollnone | | | -0.1476 | | 0.7764 | | -0.190 | |  |
| heatcat26:pollnone | | | -0.7360 | | 0.7815 | | -0.942 | |  |
| heatcat30:pollnone | | | 1.3908 | | 0.7817 | | 1.779 | |  |
| heatcat34:pollnone | | | 1.8015 | | 0.8013 | | 2.248 | |  |

**G) Harvest index per plant (model estimates remain exponential transformed)**

| **Maximal model:** | exp(HI)~heatcat+poll+heatcat:poll+(1\|cage) | | | | | | | | |
| --- | --- | --- | --- | --- | --- | --- | --- | --- | --- |
| **Deletion step** | **Parameter** | **χ^2^** | | **df** | | ***p*-value** | |  |  |
| **1** | heatcat:poll | 19.354 | | 4 | | 0.001 | |  |  |
| **2** | heatcat -> 18-26, 30,34 | 1.5439 | | 4 | | 0.819 | |  |  |
| **Fixed effect in minimal adequate model** | | | **Estimate** | | **SE** | | ***t*-value** | |  |
| (Intercept) | | | 2.5858 | | 0.1195 | | 21.641 | |  |
| heatcat30 | | | 0.5274 | | 0.1481 | | 3.562 | |  |
| heatcat34 | | | 0.2125 | | 0.1481 | | 1.435 | |  |
| pollnone | | | -0.3242 | | 0.1693 | | -1.915 | |  |
| heatcat30:pollnone | | | -0.9171 | | 0.2096 | | -4.375 | |  |
| heatcat34:pollnone | | | -0.3295 | | 0.2096 | | -1.572 | |  |

**H) Non-harvest components per plant**

| **Maximal model:** | nonharvest~heatcat+poll+heatcat:poll+(1\|cage) | | | | | | | | |
| --- | --- | --- | --- | --- | --- | --- | --- | --- | --- |
| **Deletion step** | **Parameter** | **χ^2^** | | **df** | | ***p*-value** | |  |  |
| **1** | heatcat:poll | 4.1255 | | 4 | | 0.389 | |  |  |
| **2** | heatcat | 16.181 | | 4 | | 0.003 | |  |  |
|  | poll | 4.7245 | | 1 | | 0.030 | |  |  |
| **3** | heatcat -> 18-26,30,34 | 3.0787 | | 2 | | 0.215 | |  |  |
|  | heatcat -> 18-26,30-34 | 5.7226 | | 3 | | 0.126 | |  |  |
| **Fixed effect in minimal adequate model** | | | **Estimate** | | **SE** | | ***t*-value** | |  |
| (Intercept) | | | 33.645 | | 1.032 | | 32.6 | |  |
| heatcat30-34 | | | -3.429 | | 1.05 | | -3.27 | |  |
| pollnone | | | 2.936 | | 1.337 | | 2.2 | |  |

**I) Coefficient of Variation (SD/mean), (model estimates remain square-root transformed)**

| **Maximal model:** | sqrt(coeffvar)~heatcat+poll+heatcat:poll+year | | | | | | |  |  |
| --- | --- | --- | --- | --- | --- | --- | --- | --- | --- |
| **Deletion step** | **Parameter** | | ***F*** | | ***p*-value** | | |  | |
| **1** | heatcat:poll | | 0.7027 | | 0.593 | | |  | |
|  | year | | 21.044 | | <0.001 | | |  | |
| **2** | heatcat | | 0.8653 | | 0.488 | | |  | |
|  | poll | | 5.4736 | | 0.022 | | |  | |
|  | year | | 21.354 | | <0.001 | | |  | |
| **3** | poll | | 5.5081 | | 0.021 | | |  | |
|  | year | | 21.489 | | <0.001 | | |  | |
| **Fixed effect in minimal adequate model** | | **Estimate** | | **SE** | | ***t*-value** | ***p*-value** | | |
| (Intercept) | | 0.82678 | | 0.04861 | | 17.009 | <2e-16 | | |
| pollnone | | 0.07215 | | 0.03074 | | 2.347 | 0.021226 | | |
| year2012-13 | | -0.19753 | | 0.05325 | | -3.71 | 0.000367 | | |
| year2013-14 | | -0.31535 | | 0.05052 | | -6.243 | 1.57E-08 | | |

**J) Mass per bean per pod, per plant**

| **Maximal model:** | tgw~heatcat+poll+heatcat:poll+year+(1\|cage) | | | | | | | |
| --- | --- | --- | --- | --- | --- | --- | --- | --- |
| **Deletion step** | **Parameter** | **χ^2^** | | **df** | | | ***p*-value** | |
| **1** | heatcat:poll | 14.91 | | 4 | | 0.005 | | |
|  | year | 13.88 | | 1 | | <0.001 | | |
| **2** | heatcat -> 18-30,34 | 10.946 | | 6 | | 0.090 | | |
|  | heatcat -> 18-26, 30, 34 | 3.7685 | | 4 | | 0.438 | | |
| **Fixed effect in minimal adequate model** | | | **Estimate** | | **SE** | | | ***t*-value** |
| (Intercept) | | | 0.46015 | | 0.02289 | | | 20.102 |
| heatcat30 | | | 0.04519 | | 0.02034 | | | 2.221 |
| heatcat34 | | | 0.05500 | | 0.02056 | | | 2.675 |
| pollnone | | | 0.03147 | | 0.02695 | | | 1.168 |
| heatcat30:pollnone | | | -0.01379 | | 0.02910 | | | -0.474 |
| heatcat34:pollnone | | | -0.10686 | | 0.02911 | | | -3.671 |

**K) Percentage nitrogen per plant**

| **Maximal model:** | nitrogen~heatcat+poll+heatcat:poll+year+(1\|cage) | | | | | | | |  |
| --- | --- | --- | --- | --- | --- | --- | --- | --- | --- |
| **Deletion step** | **Parameter** | **χ^2^** | | **df** | | ***p*-value** | |  |  |
| **1** | heatcat:poll | 6.7102 | | 4 | | 0.152 | |  |  |
|  | year | 4.6472 | | 1 | | 0.031 | |  |  |
| **2** | heatcat | 10.123 | | 4 | | 0.038 | |  |  |
|  | poll | 0.6945 | | 1 | | 0.405 | |  |  |
|  | year | 4.5724 | | 1 | | 0.032 | |  |  |
| **3** | heatcat | 10.1 | | 4 | | 0.039 | |  |  |
|  | year | 4.6124 | | 1 | | 0.032 | |  |  |
| **4** | heatcat -> 18-26,30,34 | 6.9533 | | 2 | | 0.031 | |  |  |
|  | heatcat -> 18-22, 26-34 | 0.3529 | | 3 | | 0.950 | |  |  |
| **Fixed effect in minimal adequate model** | | | **Estimate** | | **SE** | | **t-value** | | |
| (Intercept) | | | 4.47987 | | 0.05122 | | 87.47 | | |
| heatcat26-34 | | | 0.1847 | | 0.05894 | | 3.13 | | |
| year2013-14 | | | -0.12923 | | 0.05923 | | -2.18 | | |
